# Instant Futures: an experimental study of the imagination of alternative near futures thanks to science fiction

**DOI:** 10.1007/s12124-024-09885-1

**Published:** 2025-02-05

**Authors:** Laure Kloetzer, Laurent Kloetzer

**Affiliations:** 1 Institute of Psychology and Education, University of Neuchâtel, Neuchâtel, Switzerland; 2 Collectif Zanzibar, France

**Keywords:** The near future, Imagination of the future, Psychology of imagination, Combinatory process, Symbolic resources, Pedagogical uses of science-fiction

## Abstract

Following Vygotsky's seminal work, sociocultural psychology has developed a powerful theory of imagination, considered as a process with mutual and transformative impacts with the social world. In this paper, we focus on the imagination of the future, which is an arena of special social and political contestation. We argue for integrating experimental methods into the scientific study of the *re-composition, or synthesis process*, in the imagination of the future. Provoking the imagination of the future in well-structured conditions allows for intra and interpersonal comparisons, as well as for comparisons through time. We introduce an experimental task, a "protokool", inspired by the work of a French group of science fiction writers, “le collectif Zanzibar”; we also suggest a way to analyse the data collected through this "telescope into the imagination of the future" looking at a specific process of imagining the future in dystopian and utopian ways. Finally, we present some main findings from the analysis of a corpus of 186 narratives collected in a 4-year study with Bachelor students in psychology and education. We show that the process of imagining the future is asymetrical for dystopian and utopian futures. We also point at some major patterns in these imaginations of the future, and evolutions over the four years. The research has theoretical and methodological implications for the study of the imagination of the future in sociocultural psychology.

*Esthetic education is connected above all with development of the power of imagination, understood not as the ability to think up what does not exist but as the ability (skill) to see what does exist, what lies before one’s eyes. And this is not an innate but an acquired skill, with different levels of development. The ability to see what in fact exists is not a whit more common than the ability to think subtly and deeply. As Goethe said: “What is the hardest thing in the world? To see with one’s own eyes what lies before them.”*

Ilyenkov, A Contribution to a Conversation About Esthetic Education

*Oui, c'est du déjà vécu, c'est de l'avenir "de seconde main", mais c'est de l'avenir quand même.*

Guéorgui Gospodinov, *Le pays du passé*, p.143

## The sociocultural psychology of imagination

### Contemporary perspectives

Following Vygotsky's pioneer work, sociocultural psychology has progressively defined a conceptual approach to the study of imagination. Zittoun and Gillespie's integrative theory (Zittoun & Gillespie, 2016) conceive imagination as a "process by which a person temporarily decouples his or her flow of experience from the here-and-now of his or her proximal sphere of experience". They describe this decoupling "as a loop, a little voyage to a distal sphere of experience, before looping back to the proximal sphere of experience and recoupling with the immediately present socially shared reality" (Zittoun & Gillespie, 2018, p.2).

Through different publications, Zittoun & Gillespie have consistently presented imagination as involving a three-step sequence: first a trigger, that initiate the person's uncoupling from the proximal sphere of experience; then, the use of resources "drawn from a wide range of semiotic and material elements previously internalized by the person along the life course, or present in the immediate environment, through the presence of others, the affordances of the setting, or the power of guidance of complex artefacts"; third, a "return—when the person loops out of imagining, and recouples with her proximal circumstances, a few seconds or hours older" (Zittoun & Gillespie, 2018, p.3).

Additionally, the authors offer "three core dimensions to describe the variety of imaginings in which people engage". The first dimension is *time orientation*, and the authors distinguish between orientation toward the past, the future, or alternative presents. The second dimension is *semiotic distance* of the imagining, in a continuum from concrete, everyday life experiences to generalized experiences. The third dimension is *plausibility*, which translates the degree of possibility of realization of these imaginations. In further works on collective imagination, they have highlighted its political dimensions and added two new dimensions: *centrality*, which describes how centrally controlled or distributed among numerous people the vision of the future is in a specific society, and *emotional valence*, which describes how a specific society relates in a positive or negative way to a specific vision of the future. Taking socialism in the USSR and the USA as an example, they rightly point that the valence of a specific imagination of the future depends on the social context in which it is received and interpreted.

These authors highlight the "real" consequences of imagination and expand it at the sociohistorical level: "Imagination about the future, we argue, is a central steering mechanism of individual and collective behaviour". This recursive view of the power of imagination is very consistent with Vygotsky's seminal and powerful realist theory of imagination.

### Vygotsky's Realist Theory of Imagination: multiple relations between imagination and reality

Vygotsky widely developed a realist conception of imagination, particularly in chapter II of the brochure *Imagination and Creative Activity* (Vygotskij, 1930/2022). Here Vygotsky sets out four main relationships between imagination and experience. First, the basis of creative activity is the combinatory capacity of our brain, which recomposes the elements of our experience. He asks "how this combinatory creative activity occurs" (p.55). He emphasizes that the extent to which this activity develops depends on "the accumulation of our experience" (p.55). Therefore, "the first form of relationship between imagination and reality comes from the fact that any creation of the imagination is always constructed from elements taken from reality, preserved from a previous experience of the human being. It would be a miracle if the imagination could create from nothing, or had sources of creation other than previous experience. (…) Even the most fantastic creatures represent nothing more than a new combination of elements which, in the end, have been drawn from reality and have just undergone deformations or transformations through the activity of our imagination." (p.56). Vygotsky uses the example of Pushkin's hut with rooster's feet to emphasise that "imagination always creates from materials given in reality". This has an important consequence, described by Vygotky as a fundamental law of the imagination: "the creative activity of the imagination depends directly on the richness and diversity of the human being's previous experience, because experience represents the material from which the construction of fantasy is built" (p. 57–58).

Then, the relation of imagination with reality might escape the subject's direct experience and come from the indirect experience of others. Imagination "becomes a means of broadening experience because human beings can imagine what they have not seen, they can represent through the account and description of others what they have not experienced directly in their own experience, and they are not reduced to their own narrow circle and limits. On the contrary, with the help of the imagination, he can go a long way beyond them by appropriating the historical or social experience of others (…) The result is a double and reciprocal dependence between imagination and experience. If, in the first case, imagination relies on experience, in the second case, it is experience that relies on imagination" (p.60–61).

The third form of relationship involves emotions. Our feelings color our imagination. Conversely, imagination influences our feelings. Here Vygotskij draws on the work of Ribot. He uses the example of a child who, "entering a room at dusk, mistakes a hanging dress for a stranger or for a robber who has broken into the house. The character of the robber created by the child's fantasy is not real, but the fear that the child feels, his terror, is quite real in his lived experience" (p. 64).

**Fig. 1:**
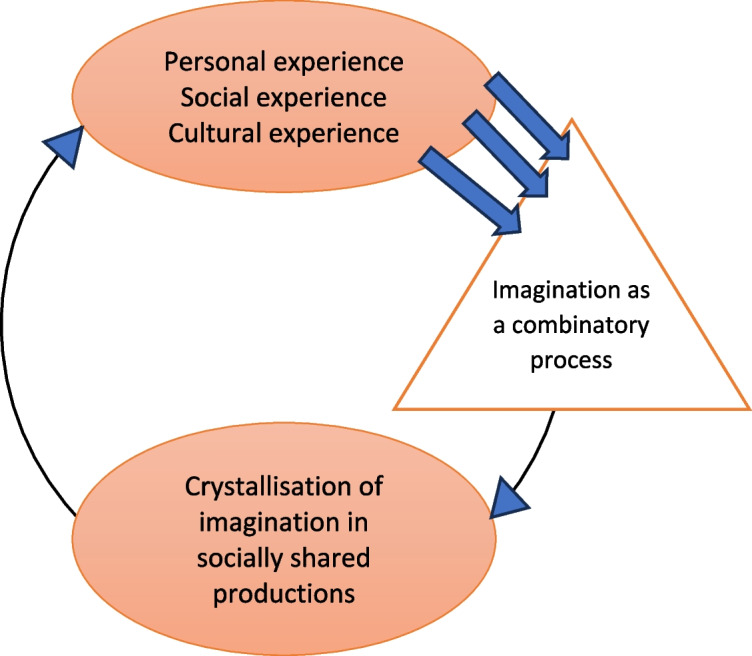
Our interpretation of Vygotsky's cycle of the imagination

The last relation refers to a fundamental dimension of the imagination, its capacity to acquire an objective existence in the real world, as crystallized imagination, in the form of a material creation or a cultural product. Vygotsky presents *the cycle of the imagination*: the imagination is inspired by real experience, and contributes to it, notably by crystallizing the products of the imagination in the objective world, which thus become available to nourish the experience of others. With Vygotsky's words: "The elements from which they are constructed have been taken from reality by the human being. From within, they have undergone a complex reworking and have been transformed into products of the imagination. Finally, by incarnating, they have returned to reality, with a new active power that transforms that reality. This is the complete cycle of the creative activity of the imagination" (Fig. 1).

The multiple connections of imagination with reality, as well as the impact of the products of our individual or collective imaginations on our personal and historical trajectories, make the specific question of the *imagination of the future* an important scientific issue, as imaginations of the futures are contested and highly political.

### Short focus on the imagination of the future

In the last ten years, the imagination of the future has been the focus of burgeoning scientific work, especially in the field of politics and migration studies. Works grounded in socio-cultural psychology have explored the dialectics of imagination and social change (Hawlina, Pedersen & Zittoun, 2020): "People’s dissatisfaction with what is, their imagining of how things once were better, or of how things may become, often supports social movements. Social movements can, in turn, bring about new imaginations for people". In this dialectical move, prolepsis (Cole, 2016) is a critical concept, i.e. the "process by which imagined futures can reshape the historical past" (Hawlina et al., 2020), as demonstrated for example in de Saint-Laurent's work on French politics (de Saint-Laurent, 2018). This echoes the notion of "imagined communities" (Anderson, 2006), which contrain the imagination of possible futures. Political imagination of the past, present and future is a critical scientific topic for a psychology of social change. Sociocultural psychologists argue that "cultural artefacts play a key role in the circulation of imagination produced by, and supporting social movements" (Hawlina et al., p.35) and that "controlling the access to cultural artefacts can systematically channel the imagination in a desired direction" (ibidem). Therefore, the centralisation or distribution of the control of the production and sharing of cultural artefacts becomes critical for our political future.

In the field of migration studies, imagination is put in relation to mobility but also immobility. For example, Cangià (Cangià, 2020; Cangià & Zittoun, 2020) studies the relations between the experience of being stuck and the possibility or impossibility of imagining an alternative future on the move. Consistently with the sociocultural psychology perspective, they conceive imagination as a dynamic process, going "from imaginaries as a socially shared and transmitted toolkit, to imagination as an ever-changing embodied and creative activity both embedded in and shaping the social and cultural world around" (Cangià & Zittoun, p.643). Zittoun (2020) explore the dialectics between the physical and imaginary trajectories of the people, showing "how trajectories of imagining enable mobility and how mobility in turn transforms people’s imagination". The authors highlight that "Imagination, as a form of symbolic mobility, represents the mental journey people can embark upon to escape the here-and-now of the present, independently from real opportunities and capacity to move" (Cangià & Zittoun, 2020).

The concept of "technologies of imagination" is also useful (Sneath et al., 2009; Pedersen, 2022).They serve as instruments of power, and can be used as a sociomaterial analytical entry into the analysis of multiple manifestations of this power (Pedersen, 2022). Analysing "how power works through the imagination" (p.161), Pedersen writes:

Technologies of the imagination can both expand or contract the imaginative horizon and are connected to the concept of imaginative resistance (Liao & Szabó Gendler, 2011), questions of power, and manipulate temporalities. Unlike symbolic resources, which are used as developmental resources (Zittoun and Gillespie 2018; Zittoun & Gillespie, 2013), technologies of the imagination are instruments of power that contain specific temporalities and are aimed at engendering certain imaginations, though they can become symbolic resources. (Pedersen, 2022, p.47).

"In other words, exploring the technologies contributes to capturing power relations and their operation. However, when combined with the sociocultural psychological approach, such an exploration also accounts for how these technologies are used and refracted through people’s experiences and trajectories" (Pedersen, 2022, p.162).

Cultural artefacts and technologies of imagination put the question of the centrality/distribution of the “power to imagine” (parallel to Spinoza's power to act), or maybe of the power to act through the process of imagining the future, at the center of the reflection. Who can imagine the future? Who is allowed to give it a try? Who is framing the content and conditions of the imagination of the future? The collectif Zanzibar aims precisely at democratizing the possibility to imagine the future.

### Research gaps and further questions

Our reading of the scientific literature points at several open problems.

First, we lack a precise understanding on how the process of creative (re)composition concretely happens, in general, and in the specific case of the imagination of the future. As noticed by contemporary authors themselves, and in accordance with Vygotsky's early thoughts on the *Psychology of Art*, this is a critical dimension: "Regarding the semiotic processes of imagining, we agree with other authors that imagination demands a complex decomposition and rearrangement of all this semiotic material, loaded with emotions and embodied experiences, into new synthesis (Vygotsky, 1933)" (Zittoun & Gillespie, 2018, p.3).

Then, we lack comparable accounts of imagining. Our understanding is that the wide range of methods favored so far (including case studies, historical studies, visual studies, analysis of diaries, video analysis of class interactions, and interviews, for example) provides very rich and valuable information about the changing connections and dynamics over time between abstract semiotic processes, social contexts and historico-material conditions in singular circumstances; however, it does not allow us to analyse a multiplicity of individual processes under similar, well-defined conditions, nor to compare them over time. We lack the possibility to compare data on *the synthesis work of imagination*, collected in similar conditions.

Finally, as both scientists and science fiction writers, we think that the scientific literature may lack some spatio-temporal precision when it treats on almost equal footing the process of imagining what I am going to have for dinner with my family tomorrow and the situation of the Empire in the year 10,191 under the reign of Emperor Padishah Shalem IV (Herbert, 1965). The difficulties of the exercise, the resources considered relevant and their creative recombination process, and the constraints taken into account may be different, for example. The future is vast, almost endless. Its temporal horizons vary from a few seconds to the end of times. In human experience, the "imagination of the future" does not exist in an abstract form (except as a scientific object of inquiry), but under concrete circumstances which deserve special attention.

Therefore, we call for *an experimental approach* in the sociocultural psychology of the imagination of the future. Although experimental studies are not a method of first choice in contemporary sociocultural psychology of imagination, they are part of the basic toolkit of developmental psychology and have a long-standing history in the field of socio-cultural psychology for the study of processes of mental development. Vygotsky famously discussed experimental findings regarding the development of higher psychological functions (Vygotski, 2014). In chapter 5 from 1939 of his book *Resolving Conflicts* (1948), Lewin discussed experiments in social contexts. He wrote:

"I am persuaded that it is possible to undertake experiments in sociology which have as much right to be called scientific experiments as those in physics and chemistry." (p.71)

His proposal is to "create set-ups which would give insight into the underlying group dynamics" (p.74–75), as he did for the study of social atmosphere as the product of specific social interactions (Lewin, 1948) or for analysing the adoption of new cooking behaviors with a social psychology approach Lewin, 1943).

The main advantage of experiments, as they are conceived in sociocultural psychology, is to *provoke the process that we want to study* thanks to the experimental task and design, and to create and document an experimental situation *whose features we can at least understand and at best control* thanks to the detailed analysis of the effects of the experimental task and experimental setting.

Recently, Perret-Clermont and colleagues have analyzed the experimental design itself (i.e., the experimental task and setting) as an intervention, which has effects by its very existence on the process under study (Tartas, Perret-Clermont & Baucal, 2016; REF). The main critic here is on the illusion that the experimental process leaves the phenomenon under study unchanged. In a sociocultural perspective, experiments are not studying de-contextualised phenemona, but concrete phenomena, produced by the process of experimenting itself. Veresov has theorized genetic-experimental method, highlighting its focus on the analysis of the process of development. He writes:

"When the analysis of things is replaced by analysis of process, then the basic problem becomes the genetic restoration of all the instances of development of the given process" (Veresov, 2004, p. 133) - "Accordingly, requirements for organisation, design and conducting the “Vygotskian experiment” differ from those which are based on classical methods" (p.135). "The design of the experimental settings should somehow lead the child to discovering the signs as external tools of solving problems and tasks given to the child. On the other hand, the experimental study should include special procedures supplying the process of transition from direct to mediated actions". (Veresov, 2014, p.144)

Working on the development of adult imagination of the future, we might take inspiration from these non classical approaches to experimental research. As stated by Valsiner, we believe that the main question should not be what the right method is, but "what research questions are worth asking, and how are methods to be derived accordingly?” (Valsiner 2009, p. 4). Our general research question concerns the re-composition, or synthesis process, in the imagination of the future. To study this process, we provoked the imagination of the future in well-structured conditions in order to allow for intra and interpersonal comparisons, as well as for comparisons through time. We introduced an experimental task, a "protokool", inspired by the work of a French group of science fiction writers, Zanzibar, and based on science-fiction writing.

## Seeing what does exist: relations of past, present and future in Science Fiction

### A "What if?" story

Our introduction of science fiction to non-experts focuses on a definition of science fiction as an artistic and narrative process grounded in the question: "What if?". What would happen, …

- if fertile women were so rare that they became the sole property of the richest men? (Margaret Atwood’s *the Handmaid’s tale*, novel and series)
- if an oppressive government decided to throw selected young people in an arena, for them to fight in public, to distract the population from their lack of freedom? (Suzanne Collins’ *The Hunger Games*, novels and films)
- if living animals had become so rare that owning a real rabbit or sheep would be a sign of social success, and companies were selling robot animals as substitutes? (Philip K. Dick’s *Do Androids Dream of Electric Sheep?*)

The work of the imagination in science-fiction writing is triggered by the process of making bold hypotheses, that may or may not be realistic, and then imagining the impact of these hypotheses on a whole social community. In this sense, science fiction relates to the future in terms of artistic possibility rather than statistical probability, unlike scientific forecasting reports such as the IPCC. This exploration of more or less realistic futures (realism here is not the point) is grounded in our experience of the present. Science fiction writers do not (obviously) live in the future and draw their inspiration from the present.

### An art of the present

As science fiction writer Alain Damasio said in an interview to a French newspaper le Point (October, 25 2018^1^):

"Science fiction is an art of the present. Of the present. Because the present is always already ample, expanded, multiple, worked from within by a myriad of vectors, thrusts, larval possibilities that science fiction has precisely the vocation to develop in order to strip away its raw tendencies. (...) It's necessarily from now on that we speak and that we go. What we sf writers always anticipate is the blowing up and blowing out of the water of a present that has been maximised to the extreme, revealed in its hidden promise, carried to the limit of what it can do (...). It is (...) an attempt to avert its sublime social perversion by exposing it".

By expanding and amplifying elements of the present which are invisible in front of our eyes, science fiction stories render them visible and allow to express and explore the creators’ own sensibility to them.

Therefore, scientists have also taken interest into science fiction as a social laboratory. Science fiction:

"provides a real space for testing different social, economic or political models and considering their consequences for individuals and societies. If we can't put a real human community "in a jar" to subject its members to different political regimes and study their reactions, we can create or study stories that contain these fictional hypotheses and offer them to fans of the genre to observe their reactions. This opens up a field of study to researchers who would not otherwise have access to it. It's as close to a laboratory as you can get. (...) For us, studying works of science fiction means looking at today's world, that of the creators as well as that of the fans of the genre. What do these works teach us about ourselves, our world and our future?" (Lacroix, 2020, Introduction, our translation^2^).

### The Near Future

As you will see in Sect. “Three Steps into the Future” below, the task given to the participants requires them to project their imagination around 30 years into the future. This is what science fiction scholars call “the Near Future”, as opposed to the “Far Future” describing worlds far beyond our horizon: the near future is a future in which the participants “may one day have to live”. As mentioned by the Encyclopedia of Science fiction in its entry *Near Future* (https://sf-encyclopedia.com/entry/near_future, entry updated on 1 May 2023).

“The near future (…) is a world which is imminently real – one of which we can have no definite knowledge, which exists only imaginatively and hypothetically, but which is nevertheless a world in which (or something like it) we may one day have to live, and towards which our present plans and ambitions must be directed. The fears and hopes reflected in our images of the near future are real, however overpessimistic or overoptimistic they may seem. In order to plan our lives we must all possess such images, and the fact that they are fictions does not mean that they are unimportant. Literary representations of the near future both reflect and nourish those images.”

The stories produced in the context of this study are then describing a future deeply rooted in the present, implicitly saying: the world will be like it is now, except for those changes.

### Science fiction and the rupture of our perception of past as a reference for the future

The relations of the future to our present and past are rooted in a historico-cultural context, and have changed through time. In her foreword to the anthology Escales 2001 (2000), the French Science fiction author Sylvie Denis reminds us that the Science Fiction genre is inextricably linked to the reflection on technology as a driving force for the transformation of societies: "The first works of proto-science fiction appeared at the beginning of the eighteenth century, with the first industrial revolution. The first works of science fiction proper were written at the end of the nineteenth century. The genre took shape and developed throughout the nineteenth and twentieth centuries (…) The birth of science fiction as a genre is inextricably linked to the birth of ideas about the future and progress" (pp.11–12). The emergence of science fiction as a literary genre reflects the rupture between the past, and the future—more precisely, the rupture between the past as a valuable inspiration for the future, mostly linked to the critical changes of our sociocultural contexts due to the technology or "techno-science", as Sylvie Denis calls it. What appears first as a quite optimistic (although not uncritical) rupture with past times, thanks to the belief in technological progress, paradoxically turns out with Neuromancer, from William Gibson, as a way to see the future as an extension of the present. Sylvie Denis captures this idea with the notion of the "bubble of now" (our translation, discussed with the author, French original is "*la bulle du présent*"):

"The real is enormous, out of all proportion to our intelligence" (Edgar Morin). I called this 'enormous' reality the 'bubble of now' in an article devoted to The Roots of Evil (Cyber Dreams 04, October 95 DLM). (...) Neuromancer isn't just a book about hackers battling it out in cyberspace; it's also a world dominated by multinational corporations, where characters define their identities in terms of objects and brands that surround them. Keeping Brand Names was a fundamental departure from the science fiction of previous decades. In a science fiction novel, names create the world. Literally. Keeping the names of companies and brands meant that this world existed as a direct extension of our own. It implied that it worked like ours, that the rules were the same. (...) In short, doing Science Fiction, is not just integrating scientific concepts into the description of the times. You have to dare to go beyond that. You have to integrate those concepts in order to transcend them. In short, you have to dare to step outside the bubble of now. But it's not easy. It requires both a sense of perspective on the times we live in and to take responsability for the vision we have of them. (Denis, 2000)

Sylvie Denis therefore uses the idea of "the bubble of now" in a dual way: first, to highlight the continuity of our imagination of the future with our vision of a present rich of multiple possibilities; second, with a critical perspective, as our responsability to go beyond this bubble of now and foster a vision of the future which is not limited to the contradictions of our current reality.

## *Three steps into the Future*: our telescope for a sociocultural, experimental inquiry into close, alternative futures

### Zanzibar and the Protokools

Our telescope into *the work of the imagination of the future* is based on a creative approach developed by a group of French science fiction writers, called "*le collectif Zanzibar"*.

According to its manifesto (https://www.zanzibar.zone/), Zanzibar aims to *"disincarcerate the future",* i.e. to free our imaginations from the ready-made (and usually frightening) visions of the future produced by established powers (multinationals, the army or the big audiovisual production studios, for example):

**Table:** 

*Zanzibar Minifeste on* https://www.zanzibar.zone/
*Despite the foresight tools and futurology consultancies of the major companies,* *despite the omnipresent talk of tomorrow being the same as today, as yesterday, or simply not being,* *we remain convinced that our futures—common and individual—belong to us,* *and that we have the power to imagine them, play with them, experiment with them and build them as we see fit.* *We are a collective of science fiction writers.* *We see our texts as places where we can meet, think and begin to disincarcerate the future.*

Zanzibar criticizes the centralisation and standardisation of the imagination of the future, offering images of the futures largely controlled and displayed by major political and economical powers. Science fiction authors are often considered as "experts of the future". However, in order to free the imagination of the future (as a cultural production, available to all) from this centralized, pre-imagined futures, Zanzibar does not focus on its own productions, but on supporting a distributed process of imagining, offering a new "technology of imagination" (Pedersen, 2018), in the form of short science fiction writing exercises, called "*Protokools*".

Protokools that have been previously imagined and used by some Zanzibar authors are gathered in the so-called "*Grand Livre des Protokools*" (The Great Book of Protokools), available online under a Creative Commons license. In this book, the Protokool that we are using here as an experimental task for exploring the work of the imagination of the future is called: *Our fears and our hopes*, or *Three Steps into the Future*.

## The Protokool "Three Steps into the Future"

Protokools are writing games. The protokool *'Three steps into the future'* is, in our experience, one of the most robust and useful writing game of our collection. It instructs participants to connect with a known, usually beloved, specific place, which they then project in their imagination into a near future (usually 30 years). It has two parts, and goes as follows:

**Table:** 

**Protokool: *Three Steps Into the Future***
**Protokool **#**1** Choose a place that you know well personally. Describe this place and what people do there 30 years in the future (in 2053), depicting a future that you don't think is desirable, that you wouldn't like to live in. After writing, those who wish to can share their text/ideas. **Protokool **#**2** Take the same place, still 30 years in the future. This time, write a ten-line text describing this place in the future, depicting a future that you think is desirable, one in which you would like to live. After writing, those who wish to can share their text/ideas.

So, the experimental task—the protokool—invites the participants to *choose a specific place*, connected to their personal experience, then to create *an undesirable version* of that place, and finally *a desirable version* of the same place. The protokool is structured in 10 min of individual writing, 10 min of collective reading and sharing, 10 min of individual writing and 10 min of collective reading and sharing again. In total, the whole exercise should take no more than 45 min.

Here the experimental task serves as a trigger into the process of imagining the future. It also constraints the way it is done. We decided to call the imaginations of the future produced "*Instant Futures*", because the participants have a very short time to think and write. The exercise, therefore, does not engage the participants into a deep elaboration of the future, nor does it provide a framework for the collective creation of the future, but it triggers feelings, images, associations, fears, hopes, dreams and desires, that are spontaneously present in the minds of the participants.

Starting with an undesirable version of the future of a beloved place allows the participants to express their deep personal fears about the future. The reading and sharing that follows often shows them that these fears are widely shared by their peers—and helps them to identify and confront them. The production of a desirable version of the same place calls for their hopes, dreams and desires. The second moment of reading and sharing allows for a collective identification of convergences, emerging paths, as well as their original contribution.

### Implementation of the experimental task in a University course: tuning the telescope to its concrete conditions of implementation

Although Zanzibar may use the same protokool in different situations and with different participants (as it is the case with the protokool *Three Steps into the Future*), the concrete use of a specific protokool requires to tune the instruction to the topic, goal, and participants. In our case, we used this protokool as some playful and reflexive part of a University Course in Environmental Psychology for Bachelor Students studying Psychology and Education. We will not discuss here the full structure of the course, nor our pedagogical motivations for using science fiction, nor the pedagogical outcomes of this tool in this course, which will be the subject of another paper.

However, the topic of the course and profile of the students might be interesting to bear in mind when reading the findings. The course is elective, so the students have chosen to study this topic. We might expect that among all students in Psychology and Education, the participants have a special interest for and awareness of environmental issues, climate change and ecology. Therefore, our telescope will not teach us about the process and products of the imagination of the future in general (something that, according to us, does not exist anyway). But it will help us look at how the imagination of the future proceeds, for different people, in the specific conditions that we have provoked.

These conditions can be defined as follows:

- due to time constraints (*10 min*), people will imagine *Instant Futures* (Like Instant Noodles, Instant Futures unfold by pouring a little hot water over them), i.e., they will display rather spontaneous, unsophisticated imaginations of the future;
- due to the instruction ("*30 years from now"*), they will imagine close futures, what science fiction experts call Near Future (see above);
- due to the instruction ("*Choose a place that you know well personally"*), these futures will be centered around a specific place, probably with which the students have some emotional connection;
- through its instructions again ("*a future that you don't think is desirable future, a future that you think is desirable"*), the exercise calls for affects in the imagination of the future. The imaginations of the future will be forced into two contrasted versions: a utopian, and a dystopian future of the same place;
- due to the temporal design, the writing of the utopian versions will be influenced by the writing and sharing of the dystopian versions, which have been first read aloud;
- due to the topic of the elective class, we expect the students to write about humans and more-than-humans (plants and other animals).

Writing a dystopian or utopian version of the future does not entail tasks of similar difficulties. As can be seen in the professional science fiction literature, the task of imagining positive futures is much more difficult as the task to imagine negative futures. The later task requires to know what, in the present reality, is at stake, as well as what we really care for and would miss if it would be destroyed; the first task requires the same, plus to imagine a path towards our dreams. This is why we begin with the task of writing a dystopian future, as a preparation step into a projection into a desirable future.

## Analysing the data collected

### The corpus

The data collected are 176 short narratives of the future produced through the protokool "*Three steps into the future*" previously presented. The participants are Bachelor students, aged 18 to 25, with a few exceptions (older mature students) and an average age of 22 years.

The data have been collected over four years. There is no major difference between the populations of students in the four years under study, except the number of participants in the course, which strongly increased in the last year. We will provide data for each year and for the total corpus, so that the readers can get an overview of the variations between years. These variations are expected, due to the small number of texts each year.

All students whose stories have been included in our corpus have given their written consent (opt-in) after the end of the course for their stories to be analyzed anonymously for research purposes. In Table 1 below, the first number is the number of students who gave their written informed consent for the research compared to the total number of the students in the course that year (for example for the last year: 32 students gave their consent among the 36 students participating to the course).

**Table 1:** Data collected

*Three steps into the future*	Number of students agreeing for the use of their narratives for research	Instant Future: undesirable version	Instant Future: desirable version
Year 2020–2021	19/21	19	19
Year 2021–2022	19/25	19	19
Year 2022–2023	18/23	18	18
Year 2023–2024	32/36	32	32
Total	88 students, 176 narratives	88 dystopian narratives	88 utopian narratives

### Our research questions

In this paper, we focus on the analysis of *the process of imagination* of the future in a specific, provoked, playful situation. Thanks to our experimental task, we could compare the narratives (a) on an intra and interpersonal level (similarities and differences for each student and between students), (b) looking at the resources supporting the process of imagining in the utopian and dystopian versions, and (c) over time. In this paper, we will focus on the last two questions.

Therefore, our research questions are:

A. How does the process of imagining differs between utopian and dystopian versions? Which fictional resources are used to support these processes of imagination and how?

B. Does the imagination of the future in the protokool evolve over the four years of the data collection, and how?

### Our method of analysis

To answer question A above, our method of analysis is inspired by Vygotsky's realist theory of imagination previously presented. We identified **three major components** that could be re-combined in these texts.

The first is **the element of personal biography**. Personal biographical elements are called for by the instruction itself, which invites the students to *choose a place they know well*. We will analyse the choice of this place (what kind of place is it?) as well as which aspect of their life, and intimacy with this place, the students choose to highlight. The exercise explicitly mobilises affects (undesirable future, desirable future) in the construction of a narrative of the future. Therefore, affects (as well as memory and reflection) help to organise the selection of elements of experience to be reworked for the narrative.

The second is **fictional resources**, which are also called upon by the instruction itself: this creative writing exercise is explicitly presented as a science fiction game, and it follows a short introduction to science fiction as a thought experience and to some technics of science fiction writing. Therefore, we might expect students to draw inspiration from popular science fiction stories or *tropes*, which are present in films, books or video games. In our coding, we analysed separately their undesirable and desirable versions of the future, in order to identify a number of (science) fiction tropes, and we coded them in the corpus.

The third is what we call **the 'Zeitgeist'**. This category includes events from the world news as well as ideas of the time, present as social discourses or representations. This connection between this instantaneous imagination of the future and social events and discourses is visible in the changes in the short stories over the years. During the four years of data collection, we faced two major crises: the COVID 19 (in a present phase for the students taking the course in autumn 2020, and as a recent past for everyone else) and the invasion of Ukraine (in February 2022, so present in the minds and lives of the students taking the course in autumn 2022 and also in 2023).

Two independent coders read the short stories, suggested and discussed a first coding of 2 × 29 short stories (the data of the first year and the first data of the last year), and established a final coding grid for each category. Then the two authors coded the corpus for the three dimensions above. In some cases, students provided a short text analysing in their own words their inspirations in their imagination of the future. These texts don't provide a deep and systematic examination of the 'building blocks' of their short stories, so we didn't use them in our systematic coding, which focused on the corpus of the short stories themselves. We did, however, review these analyses produced by the students after our full initial coding, to see if we had missed any important elements.

To answer question B, we proceeded in a slightly different way. We focused on the desirable versions of the future. Based on our first readings of the corpus, we identified four general trends (the future offers a harmonious co-existence of humans and more-than-humans; the future offers convivial and resilient communities; the future is the continuity of the past; the future is described after the catastrophy, when all problems have been solved without any convincing explanation of the change), which we decided to systematically quantify in the corpus.

## Looking into the imagining of close, alternative futures: some findings from the use of our experimental device

We can not review all findings of the research in this paper, and have to choose some major results. First, we will show that the process of imagining the future is asymetrical for negative/dystopian, or positive/utopian futures. Then we will point at some major patterns in these imaginations of the future, and their evolutions over the four years.

### Asymetrical imagining of positive and negative futures

In almost 80% of the undesirable stories we can identify at least one element of science fictional inspiration. It has long been argued that our cultural productions of the future are full of dystopian and apocalyptic visions. It seems that these are quite efficiently infused into students' instant futures, sometimes in powerful personal re-combinations. In comparison, fictional resources lack to inspire utopias. More than 1/3 of the students did not use any fictional resource (at least that we could identify) in their writing, and only 20% used some science fictional resources, confirming the lack of popular and positive science fiction stories. The two main resources mediating the imagination of positive futures are the cultural vision of harmonious life between humans and more-than human beings (present in Western culture at least from Saint Francis of Assisi to Disney's Snow White animated movie) and the current ecological discourses, supporting respectively.

#### A. Science fiction as a cultural resource for dystopian imagination

Our repeated, careful readings and initial analyses of the corpus of 88 stories describing undesirable versions of the future led to the identification of a number of science fiction tropes, i.e. imaginary motifs that are common to several science fiction stories, such as flying cars, a polluted world, totalitarian universes that select humans on the basis of genetic technologies, a nuclear apocalypse, the rationing of vital resources, and so on. These motifs are often combined in systematic ways that echo their use in dominant fictions.

Our seven categories try and capture the (science) fictional inspirations present in the students' stories: five of them are labelled by popular science fiction works that collect some of these common combinations. The students's stories do not faithfully reflect the fictional works from which they take inspiration, but integrate common patterns of this universe. The other two categories are 'none' (when we found no visible science fiction trope) and 'other' (specific, rare, fictional influences). The names we chose for the categories are therefore somewhat arbitrary, selected as representatives of their category, they help the reader understand what the students are talking about, rather than refer to any particular book or film.

Here are our final seven categories:

0. No visible (science) fiction trope

1. *Wall-E trope*: a dead world, without nature, no living being, especially plants and animals, all is full of concrete, waste and pollution

2. *The Road trope*: nuclear apocalypse, with or without a post-apocalypse life

3. *Blade Runner trope*: a world of machines and androids, humans transformed into machines, intelligent computers, robots everywhere

4. *Brave New World trope*: a highly hierarchical and elitist society, with resources distributed according to one's status, dumbing down of the masses and genetic manipulation

5. *Mad Max trope*: the struggle for survival in small, scattered communities, and the cornering of resources

6. Other inspirations (for example: the French movie le *Règne animal*, in which some humans transform themselves progressively into animals).

The dystopian futures present strong similarities (Table 2). Pollution, leading to the death of more-than-human beings, is the most common element present in these dystopia. 1/3 of the students used, one way or another, the Wall-E trope, i.e. described a dead world, in which no non-human living being—especially plants—can be found alive, or only in a mutant form, due to pollution. We find a good example of this trope in the following stories:

By 2052, this area once rich in flora and fauna will have disappeared. The water in the lake will be polluted with chemicals. This place that was so beautiful every season will now be grey and lifeless. The trees will be dead, probably replaced by factories dumping their products into the lake. Animals will be driven out of their territory or die from the toxicity of the smoke coming out of the factories. This once beautiful landscape will no longer exist.

*Example 1(Student protokool for the undesirable future, 2020, P18)*

**Table 2:** Identifiable science fiction tropes in undesirable futures (nb—percentage) (Of course, the pourcentages do not make much sense with small numbers of participants, but we provide them because they make sense for the total population and make understanding clearer)

Identifiable science fiction tropes	2020	2021	2022	2023		total
1. None	6—32%	5—26%	3—17%		6—19%	20—23%
2. Wall-E trope	7—37%	3—16%	6—33%		11—34%	27—31%
3. The Road trope	1—5%	3—16%	1—6%		1—3%	6—7%
4. Blade Runner trope	2—11%	4—21%	2—11%		5—16%	13—15%
5. Brave New World trope	1—5%	3—16%	3—17%		4—13%	11—13%
6. Mad Max trope	1—5%	0—0%	2—11%		4—13%	7—8%
7. Other	1—5%	1—5%	1—6%		1—3%	4—5%

This vision of a dead world dominated by pollution can be combined to other fantastic ideas, for example communities fighting for scarce resources under the ideological dominancy of new powerful characters, like in the following story:

In the not-too-distant undesirable future, Lake Neuchâtel has almost completely dried up. The survivors, under a blazing sun and surrounded by desertifying Mediterranean vegetation that still consists of a few olive trees, have to hide from the unpredictable sandstorms that frequently sweep through the region. Since the Great Conquest of War, Famine and Plague, food and water have become the most precious commodities, second only to the quest for eternal salvation granted by the High Priestess. She lives upstairs in the castle, surrounded by a court hungry for the last vestiges of power and with an iron grip on the production and storage of the only resources available. She is also in charge of Sunday Mass, where she preaches the duty of restriction and polygyny for the few males still able to reproduce despite the consequences of hyper-hormoned waters and exposure to the various waves and pollution that have wiped out most ecosystems.

*Example 2 (Student protokool for the undesirable future, 2023, P28)*

#### B. The fictional resources of utopias

Proceeding in a parallel way, we identified fictional resources for the construction of desirable futures (Table 3).

**Table 3:** Identifiable science fiction tropes in desirable futures (count – percentage)

Identifiable science fiction tropes	2020	2021	2022	2023	total
1. None	9—47%	8—42%	8—44%	7—22%	32—36%
2. *Green world* discourse	8—42%	5—26%	5—28%	9—28%	27—31%
3. *Snow White* trope	1—5%	2—11%	2—11%	7—22%	12—14%
4. *Nausicaa* trope	0—0%	1—5%	0—0%	5—16%	6—7%
5. *Star Trek* trope	1—5%	0—0%	0—0%	3—9%	4—5%
6. Other	0—0%	3—16%	3—17%	1—3%	7—8%

The main finding is the lack of fictional resources for positive futures (more than 1/3 of the stories).

When cultural resources can be identified, they refer mostly to the fictionalisation of already existing models—resulting in what we call the *Green World discourse*—i.e., the (fictional) idea that everyday ecology (reduced consumption, organic farming, vegetarianism, renewable energies, soft mobilities…) is established as a shared social reality (1/3 of the stories). Additionally, one powerful image inspires 14% of the stories: tracing back to the visions of the Eden Garden, and their expression for example in the discourse of Saint Francis to Brother Sun and Sister Moon, they talk about harmony between all living beings, humans and more-than-humans. This powerful image is echoed by the animated movie Snow White, by Walt Disney, for example, as interspecies harmonious life and empathic communication in the forest, and this is why we decided to call this fictional resource the *Snow White* trope. The inspiration is taken from a-historical utopias (dreamed past or fairy tales). Finally, we could identify some rare fictional inspirations integrating contrasted kinds of technology. On one side, in what we called the *Nausicaa* trope, technologies are ambivalent, and can serve as ways to destroy but also restore respectful ways of life with more-than-human beings. On the other side, in the *Star Trek* trope, one powerful technology brings immense beneficial effects to humanity. To conclude, some students mentioned unique inspirations, like *The Dispossessed* by Ursula le Guin, or the French movie *La Belle Verte*, in which an ambassador from the Green Planet tries to convince humans living on Earth to transform their behavior and evolve toward a more caring and ecological society, thanks to her psychic powers.

Here are the tropes that we coded:

1. No visible fictional resource
2. *Green World* discourse: a world in which everyday ecology is a social reality
3. *Snow White* trope: interspecies harmony of life in pristine nature
4. *Nausicaa* trope: an utopia of life in peace, but with ambivalent technologies
5. *Star Trek* trope: a powerful technology with beneficial effects
6. Other unique inspirations (The movie *La Belle Verte*, *The Dispossessed* by Ursula le Guin…).

Below you can read an extreme version of the *Snow White* trope, in which animal communication is pushed towards kinship:

''Let your thoughts pass as if they were clouds. Breathe in and out. Focus your attention on your energy centres. Breathe in and out. Let the light in...''

Today I'm having trouble concentrating and clearing my head. My meditation feels like it's been going on for hours and my teacher Jocelyne's voice can't calm me down. I'm too excited about my ceremony tomorrow and my thoughts are racing. Luckily, I hear Moustachus, the cat, finally scratching at the classroom door, signalling the end of class. At last! I get up quickly, wave to Jocelyne, give Moustachus a little cuddle and run to my new electric unicycle. My parents gave it to me for a birthday present, and it runs on the photosynthetic energy of these magnificent reconstructed forests.

I speed off to the big golden tree to meet up with my great friend and adviser Slipper, my guardian angel who happens to be a spotted hare. He chose me when I was two. Our guardian must appear in our lives before we're five, or it means we'll remain katchis until the end.

I know I was once a katchi in another life, our shaman and neighbour Karla told me so. Soon I'll even be able to recall memories from that life, but we'll have to wait a little longer. We can't disperse our spiritual energies too quickly, or they can turn against us, she told me with her serene presence. So I calmly waited for the moment.

Today he's going to give me all the advice a guardian needs to give before tomorrow's ceremony. The ceremony where our superpowers and our role in society will be announced.

*Example 3 (Student's protokool for a desirable future, 2023, P23)*

Here an interesting "Star Trek trope" story, in which a student imagines a technological innovation, the "créacier", or *glacier maker*, which allowed to recreate all melting glaciers of the Alpine mountains and solve the water crisis:

Valais: 2053. We have finally solved the water problem. The Rhône Valley has regained all its splendour. Since the authorities installed the créacier - our greatest invention, capable of creating glaciers even in the most austere climates - the Rhône glacier has reappeared. It once again feeds the entire valley with clear, abundant water. Animals have regained all their rights in this body of water. Before the creacier was set in motion, we feared civil war over this priceless resource. But instead, we've rediscovered the joy of living in community with all the towns along the river. The flow of the Rhône even provides the canton of Valais and the canton of Vaud with green electricity. But this was only the beginning. The authorities are going to introduce the creacier in other regions of our country before exporting it throughout the world, so that water is no longer a daily struggle for the survival of all living beings.

*Example 4 (Student's protokool for a desirable future, 2023, P11)*

### Evolution over time: trends in desirable futures

This section explores the evolution of some patterns frequently identified in desirable futures over the four years of the course.

#### A. Trends in desirable futures: A combination of social and interspecies harmony

First, we find the stability over time of two patterns, which are frequently associated in the stories of the students: the harmony with nature, as previously discussed, is frequently combined with social harmony—the harmony of social relations in inclusive human societies. These two common aspirations of the students in this course are strongly co-present in their stories (Tables 4 and 5).

**Table 4:** Harmony with nature in desirable futures

Harmony with nature in desirable futures	Frequency (total corpus)	*2020*	*2021*	*2022*	*2023*
No	7%	5%	5%	6%	9%
Maybe	13%	21%	0%	17%	13%
Yes	81%	74%	95%	78%	78%

**Table 5:** Inclusive and supportive human societies in desirable futures

Inclusive and supportive human societies in desirable futures	Frequency (total corpus)	*2020*	*2021*	*2022*	*2023*
No	5%	11%	5%	6%	0%
Maybe	31%	26%	53%	22%	25%
Yes	65%	63%	42%	72%	75%

This double harmony takes different forms, from dream images of bucolic life in the woods to more realist accounts. In the example below, the student takes into account the reality of climate change. Adopting a subjective, first-person view, he describes the reflection of an inhabitant of Valais in 30 years. His desirable version combines strategies for water management in the Valais (a central problem strongly emerging today), rehabilitation of local landscapes (wooded pastures), sustainable agriculture, and ecological local transports, in a convincing future in which humans and nature work together to face the challenges of global warming:

"8°C for February is not too bad. Last year the average was 11°C. It's even a bit chilly for cycling up the valley. And maybe I'm a bit old for such a feat. Fortunately, the commune of Sion et Vallées will soon have finished building the cable car that will take you up to Arolla in 1h30. So it wasn't such a bad idea to collectively buy the dam so that all the surrounding communes could benefit. Once we're connected to the Rhône Valley again, we'll be able to cultivate the hillsides more efficiently. Of course, the species present will have changed, but our methods allow for interesting synergies between biodiversity and sustainable forestry and livestock farming. And we'll finally be able to get government subsidies for creating wooded pastures! So we've seen worse ends of the world. Well, I can't dawdle, I have to be at the scree management meeting at 10am. The geologist from Lausanne and a group from Vallee d'Aosta are coming on purpose, it would be a shame to miss them".

*Example 5 (Student's protokool for a desirable future, 2022, P9)*

#### B. Trends in desirable futures: The "nothing has changed" pattern

The most common trend that we identified in the stories of desirable futures is the pattern that we could call "the future is like the past". (Table 6) More than half the stories fall into this pattern. It consists of imagining the future as a continuity of a happy (dreamed) past, more precisely as the reproduction of some happy childhood memories. In this case, the desirable futures are a "nothing as changed story", as in the prototypic example below:

**Table 6:** Trend "the future is like the past" in desirable futures

*The future is like the past (desirable futures)*	*Frequency* *(total corpus)*	*2020*	*2021*	*2022*	*2023*
No	31%	16%	21%	22%	50%
Maybe	15%	16%	26%	17%	6%
Yes	55%	68%	53%	61%	44%

There are only a few farms and farmers on this mountain. The grass is so green and the air so clean. These are the same lands that my ancestors farmed and nothing has changed. Every corner is connected to our childhood memories. I hope this place stays the same.

*Example 6 (Student's protokool for a desirable future, 2020, P7)*

#### C. Trends in desirable futures: The dismissal of imagination?

Finally, we have identified a pattern which we decided to call "after the catastrophy, all is fine". In this pattern, the students project a future in which all problems have been solved and are behind us, without any attempt to imagine a convincing path towards this new situation, which is a return to old times (Table 7).

**Table 7:** Trend "after the catastrophy" in desirable futures

After the catastrophy, all is fine (desirable futures)	Frequency (total corpus)	*2020*	*2021*	*2022*	*2023*
No	66%	89%	74%	50%	56%
Maybe	9%	5%	11%	17%	6%
Yes	25%	5%	16%	33%	38%

Here is an example of the "after the catastrophy" trend. The student projects herself as a teacher at the University of Neuchâtel in 30 years, discussing the latest IPCC report with her students in 2053:

2053, on the stage of the Aula des Jeunes rives at UniNe. I was far from imagining what human beings are capable of, and too often I underestimated them. How many times were we told during our studies that the climate crisis had reached the point of no return, that social inequalities were getting worse and worse and that our future in this world was highly questionable. I can't count the number of times we were told that saving the planet, restoring peace and bringing justice to the most disadvantaged would require such a massive effort that it would be almost impossible. But never say never.

On this 26 October 2053, it is with great emotion and joy that I read to my students extracts from the latest IPCC report, which is (for once) optimistic and encouraging. The major powers have finally acknowledged their involvement in the climate crisis and decided to take concrete action. The list is far too long to go into, but suffice it to say that we're there! We have found solutions to mitigate the consequences of the climate crisis, to preserve our environment and, above all, to ensure that this does not happen at the expense of large numbers of people. It wasn't hoped for, but I'm proud to be able to hope for a future for my grandchildren.

*Example 6 (Student's protokool for a desirable future, 2023, P20)*

In this example, trying to answer our instruction to write about a desirable future, the student says "I'm proud to be able to hope for a future for my grandchildren", but does not try and express any path towards this achievement: how the society has managed to solve the ecological and climate change crisis remains a mystery, except for the mention: "The major powers have finally acknowledged their involvement in the climate crisis and decided to take concrete action. The list is far too long to go into, but suffice it to say that we're there!".

This trend could be interpreted as a loss of the ability of imagining the path toward a better future—a kind of surrender of our imagination. It could even be seen as the progression of despair, i.e. the uncapacity to see how things could go better. This paradoxical view combining surrender regarding our capacity to solve the situation, and still some (provoked) hope regarding humanity, results in the projection in a happy and healthy world *after the catastrophy has been solved*—but without any attempt to imagine a realistic or fantastic pathway toward this happy ending of the scary story. According to our data, this pattern is in constant progress among our students: it can be identified in 5% of the stories in 2020, 16% in 2021, 33% in 2022, and 38% of the stories in 2023.

## Discussion on the process of imagining the future

Our research allows us to document the work of imagination, in a specific task, which requires instant imaginations of the future and their circulation in a specific community, for a specific population (Undergraduate students in Psychology-Education in French-speaking Switzerland). We have been looking at the process of rec-combination and synthesis in the imagination of the future. Consistently with Vygotsky's theory on imagination, we have analyzed the productions of our telescope into the imagination of the future (the Protokool X, Three Steps into the Future) with a three components model.

This model highlights the elements of the students' experience which are combined to create these instant futures: biographical elements, fictional resources available in the culture, and elements of the "Zeitgeist" (ideas, events which are present in the contemporary social discourses). In this paper, we focused on the fictional elements.

This exercise shows that imagining the future is difficult, and processes differently for an undesirable future and for a desirable future.

The undesirable future is based on our fears. It is strongly characterised in the students' instant imaginations by pollution, the death of non-humans, trees or animals, the loss of rich and embodied human relationships, the commodification of nature, and the destruction of the places we love, especially those linked to childhood.

The desirable future is based on our hopes. Half of the projections (55%) are very conservative, promoting the continuity of what is, or a fantasized return to what was. 81% of desirable futures in this population feature harmonious relationships with nature, which is not a surprise in this community. 65% present inclusive and supportive human communities. Organic farming, local consumption, caring and democratic communities, slow mobility, renewable energies, education by nature, are present in numerous stories. This shows that a set of common ecological ideas, already present in our society, nourishes the imagination of this students' community, and help them draft pathways towards futures they would like to live in.

Our analysis confirms the lack of positive fictional resources for imagining desirable futures. The (science) fiction tropes that can be identified in the students' stories come from various dystopian visions of the future that meet and feed the students' anxieties (about pollution, the destruction of nature and human communities). Few fictional tropes can be identified in the positive versions of the future.

In a superficial reading of the desirable futures drafted by our students, they could be qualified as 'rather poor'. However, if we consider closer the process of imagining positive futures, our reading might change. The attachment of the students to their childhood memories reveals that their past and present life is quite happy—in Switzerland, the anxiety of the future is not linked to the material conditions of existence (which could appear as a dreamed future for a lot of young people in poorer countries), but to our growing awareness of its immense cost for the planet, and therefore unsustainability for the future. So, the anxiety mostly comes from our global and personal consciousness of the impossibility to continue our life as it is. Faced to this crisis of ways of life, linked to a pressing awareness of its negative aspects, the ways to move forward into a positive future are limited: on one side, Swiss young people can try and reduce this crisis of consciousness either by actively denying the climate and ecological crisis, or by going back to times in which this climate and ecological crisis was out-of-scope for most people, i.e. in the past. This might explain the fantasized conservative dreams of a return to happy childhood memories and a harmonious life in and with unspoiled nature, expressed by half of the students. On the other side, they can try and imagine a different reality, in which our ways of life are consistent with what we know about the reality of the climate and ecological crisis. However, the idea of transitioning to a completely different society comes up against the many impossibilities that immediately spring to mind: our apparently locked-in economic and political systems make such a transition unlikely. With some historical consciousness of the huge cost of revolutions, students might prefer to draft a third way, and decide to do (in imagination) what seems feasible: navigating the zones of experimentation of alternative models that already exist in our society. Their imaginations of the future therefore often appear as a collection of small ideas that are already here: including participative farming, agroforestry, tinkering and repair, bartering and service exchange networks, biking, and democratic popular assemblies, for example. They do, in imagination, what they already do (or would like to do), which is to navigate the margins, and contribute to experimenting alternative ways of life, but in a more systematic way. Finally, in these positive futures, the *'and if'* call of science fiction does not appear as a new working hypothesis, but as *the inversion of what is at the center, and what is at the periphery*: the margins become the dominant model in their instant utopias.

A final finding, however, worries us much more than what this superficial reading could describe as the lack of ambition of the desirable versions of the future produced by our students in the short time of the protokool exercise. We noticed an emerging movement that we called "after the catastrophy", and qualified as *the surrender of our creative imagination to draft positive futures*. We are very concerned that this trend is fast and constantly growing: from 5% of the narratives in 2020, to 38% in 2023. If this continues, it will strongly signal a fundamental rupture in our potential connection to our future.

## Conclusion

Following the claims of the Zanzibar collective, the imagination of the future is too essential to be left to major political, economics or military powers with their ubiquitous mastery of technologies of imagination. It is an arena of strong social and political contestation and experimentation, with recursive effects on our sociocultural reality—and therefore a scientific object of critical importance. In this paper, we have raised three initial concerns: the lack of scientific research on the core process of the imagining of the future, which is the creative recombination of past experience and cultural resources; the lack of methods allowing intra, inter and longitudinal comparisons of the imagination of the future in controlled and comparable situations; and the lack of precision of the object "imagination of the future" itself, seen as an abstract semiotic process freed from the constraints of concrete contexts. To partly answer these concerns, we have suggested an experimental approach of the field of the imagination of the future. We decided to focus on the work of the imagination, i.e. the creative re-combination of elements taken from experience and cultural resources. We approached this creative synthesis process in an indirect way, through comparisons. We aim at producing imaginations of the future, that can be compared as they are produced in well-defined, comparable circumstances. This Vygotskian, non classical experimental approach aims at provoking, triggering, specific imaginations of the future, in order to study them.

We took inspiration from science fiction. A French group of science fiction writers, the collectif Zanzibar, has built in the last ten years some writing games, so-called "protokools", conceived as playful tools to engage non specialists into structured imaginations of our future. The protokool Three Steps into the Future serves as our telescope into the process of imagining the future. In this paper, we present this telescope and also a way of analysing the data that it allows us to produce and collect.

Our findings highlight the asymetry of the process of imagining undesirable, dystopian futures, and desirable, utopian futures, as well as stable and emerging patterns in the imagination of the future over the four years of the course. We believe that it demonstrates the interest of collecting comparable data regarding the imagination of the future.

Further research is of course needed. First, with the same tool. Further research would be welcome with the same task, and different populations. Second, with other tools. Our telescope is not a generic tool, it shows only a part of the sky. Other experimental tasks could be designed, looking at different parts of the sky. Creative methodological development will also be required to better approach the synthesis process itself, which we have approached here indirectly, through a comparison of productions.

Then, we must admit that we are worried by our disturbing final result concerning the recent and constant rise of what we have called the "after the catastrophy, all is fine" pattern. We analysed it as the dismissal of our creative ability to draft positive futures. Urgent examination of whether this trend does exist and how much in other populations would be much needed.

Finally, at the end of our research, considering the lack of fictional resources for the imagination of desirable futures reported in our research, the question remains on what could be done, on an artistic and cultural level, to support positive imaginations of the future. Dystopias are easier to write (and sell) than utopias in science fiction, for many reasons linked to the very nature of stories (see for example Bruner, 2003). By what they decide to show and destroy, dystopias may also offer very fascinating insights into positive futures. However, based on our analyses, we can argue that popular positive fictions (sometimes called *solar punk* science fiction) are much needed as they could offer more direct, collective fictional resources supporting our individual and collective capacity to imagine desirable near futures. Based on our findings, we can assume that these versions of better futures could build on the simultaneous call for respect for more-than-humans and convivial human communities, as well as expand what is already modestly present in the margins of our societies, to deploy full-fledged alternative societies.

## Data Availability

The corpus of students' narratives can be accessed upon request to the contact author (Prof. Laure Kloetzer, laure.kloetzer@unine.ch). All students, whose data have been included in the study, have given their written agreement for the use of their writings for research purposes.

## Footnotes

1 French original (Damasio, 25 octobre 2018, journal Le Point): "La science-fiction est un art du présent. Des présents. Parce que le présent est déjà toujours ample, dilaté, multiple, travaillé du dedans par une myriade de vecteurs, de poussées, de possibilités larvaires que la science-fiction a précisément vocation à désenvelopper pour en désosser les tendances crues. (…) C'est nécessairement de maintenant qu'on parle et qu'on part. Ce que nous anticipons, nous, auteurs de SF, c'est toujours la mise hors d'air et hors d'eau d'un présent potentialisé à l'extrême, révélé dans sa promesse cachée, porté au bout de ce qu'il peut. (…) C'est (…) tenter de conjurer, en l'exposant, sa sublime perversion sociale." (*Le point*, https://www.lepoint.fr/pop-culture/alain-damasio-la-science-fiction-est-un-art-du-present-25-10-2018-2265889_2920.php).

2 French original version: La science-fiction "incarne un véritable espace pour tester des modèles sociaux, économiques ou politiques différents et en envisager les conséquences sur les individus et les sociétés. Si on ne peut pas mettre dans un bocal une communauté humaine réelle pour soumettre ses membres à différents régimes politiques et étudier leurs réactions, on peut créer ou étudier des récits qui mettent en scène ces hypothèses fictives et les proposer aux amateurs du genre pour observer leurs réactions à eux. Cela ouvre un espace d'étude pour des chercheurs auxquels on ne pourrait avoir accès autrement. C'est là le plus près que l'on puisse être d'un laboratoire. (…) Pour nous, étudier les œuvres de science-fiction revient à se pencher sur le monde actuel, celui des créateurs autant que celui des amateurs de genre. Que nous enseignent ces œuvres sur nous, sur notre monde et sur notre devenir ?" (Lacroix, 2020, Introduction).

## Declarations

### Clinical trial

Clinical trial number: not applicable.
